# Learning curves for point-of-care ultrasound image acquisition for novice learners in a longitudinal curriculum

**DOI:** 10.1186/s13089-023-00329-2

**Published:** 2023-07-05

**Authors:** Mike Breunig, Andrew Hanson, Michael Huckabee

**Affiliations:** 1 Division of Hospital Internal Medicine, Mayo Clinic, 200 First Street SW, Rochester, MN 55905 USA; 2 Quantitative Health Sciences, Mayo Clinic, 200 First Street SW, Rochester, MN 55905 USA; 3 Mayo Clinic PA Program, Mayo Clinic School of Health Sciences, 200 First Street SW, Rochester, MN 55905 USA

**Keywords:** Point-of-care ultrasound, POCUS, Learning curves, Medical education, Novice learners, Physician assistant

## Abstract

**Background:**

A learning curve is graphical representation of the relationship between effort, such as repetitive practice or time spent, and the resultant learning based on specific outcomes. Group learning curves provide information for designing educational interventions or assessments. Little is known regarding the learning curves for Point-of-Care Ultrasound (POCUS) psychomotor skill acquisition of novice learners. As POCUS inclusion in education increases, a more thorough understanding of this topic is needed to allow educators to make informed decisions regarding curriculum design. The purpose of this research study is to: (A) define the psychomotor skill acquisition learning curves of novice Physician Assistant students, and (B) analyze the learning curves for the individual image quality components of depth, gain and tomographic axis.

**Results:**

A total of 2695 examinations were completed and reviewed. On group-level learning curves, plateau points were noted to be similar for abdominal, lung, and renal systems around 17 examinations. Bladder scores were consistently good across all exam components from the start of the curriculum. For cardiac exams, students improved even after 25 exams. Learning curves for tomographic axis (angle of intersection of the ultrasound with the structure of interest) were longer than those for depth and gain. Learning curves for axis were longer than those for depth and gain.

**Conclusion:**

Bladder POCUS skills can be rapidly acquired and have the shortest learning curve. Abdominal aorta, kidney, and lung POCUS have similar learning curves, while cardiac POCUS has the longest learning curve. Analysis of learning curves for depth, axis, and gain demonstrates that axis has the longest learner curve of the three components of image quality. This finding has previously not been reported and provides a more nuanced understanding of psychomotor skill learning for novices. Learners might benefit from educators paying particular attention to optimizing the unique tomographic axis for each organ system.

**Supplementary Information:**

The online version contains supplementary material available at 10.1186/s13089-023-00329-2.

## Background

A learning curve is graphical representation of the relationship between effort, such as repetitive practice or time spent, and the resultant learning based on specific outcomes [1]. Learning curves align with an important learning principle that practice improves performance and “sufficient practice leads to higher levels of achievement” [1]. Practically, individual learning curves provide insight into the progression of learning for a specific student, potentially identifying students who have achieved specified learning outcomes or who require additional education or remediation. Group learning curves provide information for designing educational interventions or assessments [1]. Plateaus on learning curves represent a “spurious limit [to learning],” where further improvement in performance becomes unlikely in the given context. Plateaus may be surpassed if additional educational interventions and assessments are applied [1, 2].

Point-of-Care Ultrasound (POCUS) is a goal-directed ultrasound examination performed by a clinician at a patient’s bedside to answer a specific diagnostic or therapeutic question [3]. Use in clinical practice and incorporation into medical education programs are common and increasing [3]. POCUS involves both cognitive and psychomotor skillsets. The quality of POCUS images is largely dependent on the operator selecting the optimal depth, gain, and tomographic axis (angle of intersection of the ultrasound with the structure of interest) while manipulating the ultrasound device. Meticulous attention to these components of image quality is necessary during education and learner assessment [4]. Higher numbers of POCUS examinations performed by learners have been shown to be associated with improved learning outcomes. In a 2019 study, Duanmu demonstrated that emergency medicine resident physicians who had performed greater than 300 total POCUS examinations performed better on an observed structured clinical examination than colleagues who had performed fewer than 300 POCUS examinations [5]. While experts have advocated for 25–50 examinations per organ system, the amount of practice required is not well known [4–6].

Prior studies on learning curves for different POCUS applications reveal substantial variations in study size, study population, and study methodology. In several larger studies, learning curves of resident and attending physicians for various POCUS applications were assessed, however, these learners had variable degrees of POCUS experience, thus potentially altering learning curves and plateau points [7–9]. Despite the known benefit of a longitudinal curriculum for optimal POCUS knowledge retention, studies assessing learning curves of novices have included only a single educational intervention [10–13]. Often times, studies of learning curves include a mixed assessment of cognitive and psychomotor skillsets [8, 12, 14, 15]. This potentially limits the usefulness of the data, as learning curves for cognitive and psychomotor components of POCUS differ [7]. A thorough assessment of learning curves of the psychomotor skillset for novice learners in a longitudinal POCUS curriculum has not been completed.

A 2017 systematic review assessing POCUS training programs for novice medical learners demonstrated that while incorporation of ultrasound in medical education is feasible, areas for further research include identification of trainee learning curves [16]. The purpose of this research study is to define the learning curves psychomotor skill acquisition of novice Physician Assistant (PA) students enrolled in a longitudinal POCUS curriculum. While image interpretation and clinical integration are necessary components of POCUS training, the emphasis of this study is the psychomotor skillset and assessment of these cognitive skills the scope of this study. Additionally, we sought to explore the learning curves for the individual components of image quality include depth, gain, and tomographic axis, as this has not been previously reported in the literature. A more thorough understanding of this topic will allow educators to make informed decisions regarding curriculum design and implementation, and learner assessment.

## Methods

Twenty-two PA students were enrolled retrospectively in the study, upon completion of requirements for graduation at the {redacted} PA Program, demographic data were collected and reported. All students completed a previously described longitudinal POCUS curriculum, utilizing a flipped classroom setting with pre-class video lectures and in-class image interpretation, case studies, and proctored hands-on skill practice [17]. A multimodal evaluation utilizing multiple choice quizzes/tests, hands-on skills assessments, and a portfolio of images was utilized for the curriculum. Students were required to complete a minimum of 25 POCUS examinations each of the heart (minimum of 3 of the parasternal long axis, parasternal short axis, apical 4 chamber, subcostal 4 chamber, and inferior vena cava), lungs (minimum of 4 exam points on a hemithorax), aorta (transverse and longitudinal), kidney (transverse and longitudinal), and bladder (transverse and longitudinal) [17]. Feedback was provided after 5, 10, and 25 submitted examinations. All examinations were stored in a personalized digital archive utilizing Butterfly Network cloud software. All examinations were reviewed by a PA from the Program faculty who has completed external certification through the American College of Chest Physicians and two instructional faculty who were PAs in Emergency Medicine at the affiliated health system who have completed internal POCUS credentialing. Image quality was assessed using the American College of Emergency Physician suggested quality assurance 5-point Likert scale rubric, expanded to include individual assessments of depth, gain, and tomographic axis [18–20]. The research project was deemed exempt by the Institutional Review Board.

Descriptive statistics were reported outlining the median, minimum, and maximum number of examinations completed according to organ system and as an overall total. Examination scores were presented within each organ system both as frequency and percentage, (Table 1) and as stacked bar charts (Additional file 1: Figures S1, 3, 5, 7, and 9). Multivariable cumulative link mixed effects models were used to assess the association between number of examinations and increased odds of higher score while accounting for repeated measurements within subject. Models were adjusted for organ type, as well as month of program and number of examinations of the given organ both parameterized as a 2-degree of freedom natural cubic spline with knots at 3, 7, and 12 months and 2, 12, and 22 examinations, respectively. The interaction between organ type and the number of examinations for the given organ (parametrized as above) was also included in the model. Model results were presented visually as estimate (95% CI) for the log-odds ratio for higher score by number of examinations for the given organ compared to the first examination for the given organ. Plots were assessed visually to determine if any plateau points exist. In addition, the estimated multiplicative increase in odds (odds ratio) with 95% CI and p-values for higher scores associated with an increase of 2 examinations was presented according to the organ system at various numbers of examinations for each given organ.

**Table 1:** Rates of Likert Scale scores for overall image quality, depth, tomographic axis, and gain for all organ systems based on examination number

	Abdominal Aorta (N = 541 (%))	Bladder (N = 538) (%)	Cardiac (N = 545) (%)	Lung (N = 534) (%)	Renal (N = 537) (%)	Total (N = 2695) (%)
Depth						
1	0 (0)	0 (0)	1 (0)	0 (0)	0 (0)	1 (0)
2	0 (0)	0 (0)	1 (0)	0 (0)	1 (0)	2 (0)
3	75 (14)	27 (5)	26 (5)	6 (1)	45 (8)	179 (7)
4	180 (33)	102 (19)	148 (27)	46 (9)	159 (30)	635 (24)
5	286 (53)	409 (76)	369 (68)	482 (90)	332 (62)	1878 (70)
Axis						
1	9 (2)	0 (0)	7 (1)	1 (0)	0 (0)	17 (1)
2	15 (3)	0 (0)	15 (3)	2 (0)	1 (0)	33 (1)
3	86 (16)	20 (4)	149 (27)	56 (10)	93 (17)	404 (15)
4	113 (21)	69 (13)	188 (34)	113 (21)	187 (35)	670 (25)
5	318 (59)	449 (83)	186 (34)	362 (68)	256 (48)	1571 (58)
Gain						
1	0 (0)	0 (0)	0 (0)	0 (0)	0 (0)	0 (0)
2	3 (1)	0 (0)	0 (0)	0 (0)	1 (0)	4 (0)
3	37 (7)	10 (2)	47 (9)	12 (2)	51 (9)	157 (6)
4	144 (27)	54 (10)	213 (39)	78 (15)	135 (25)	624 (23)
5	357 (66)	474 (88)	285 (52)	444 (83)	350 (65)	1910 (71)
Overall quality						
1	6 (1)	0 (0)	3 (1)	0 (0)	0 (0)	9 (0)
2	16 (3)	0 (0)	16 (3)	2 (0)	0 (0)	34 (1)
3	89 (16)	26 (5)	118 (22)	35 (7)	93 (17)	361 (13)
4	154 (28)	90 (17)	252 (46)	120 (22)	191 (36)	807 (30)
5	276 (51)	422 (78)	156 (29)	377 (71)	253 (47)	1484 (55)

^*^Values are frequency (percentage)

All analyses were done using R version 4.1.2 (R Foundation for Statistical computing, Vienna, Austria, 2021). *P*-values < 0.05 were considered statistically significant.

## Results

All 22 students within the academic cohort were enrolled in the study. POCUS examinations were collected prospectively over the course of the two years of the program. Students were enrolled retrospectively at the time of completion of their POCUS portfolio. Sixteen students were female and 6 were male; average age at enrollment was 27 (minimum 22, maximum 40). No students had any prior ultrasound training or experience. A total of 2695 examinations were completed and reviewed: 541 of the abdominal aorta, 538 of the bladder, 545 of the heart, 534 of the lungs, and 537 of the kidneys. Median (minimum, maximum) number of examinations per organ system were 26 (21–40) for the abdominal aorta, 25 (20–32) for the bladder, 26 (22–43) for the heart, 25 (20–33) for the lung, and 26 (20–48) for the kidneys. Of all examinations, only 11% were completed on actual patients, while the remaining 89% were completed on healthy individuals (classmates, friends, significant others, or family). There were 4, 4, 3, 6, and 5 students where fewer than 25 images for abdominal aorta, bladder, cardiac, lung, and renal organ systems were noted in the data set, respectively.

**Fig. 1:**
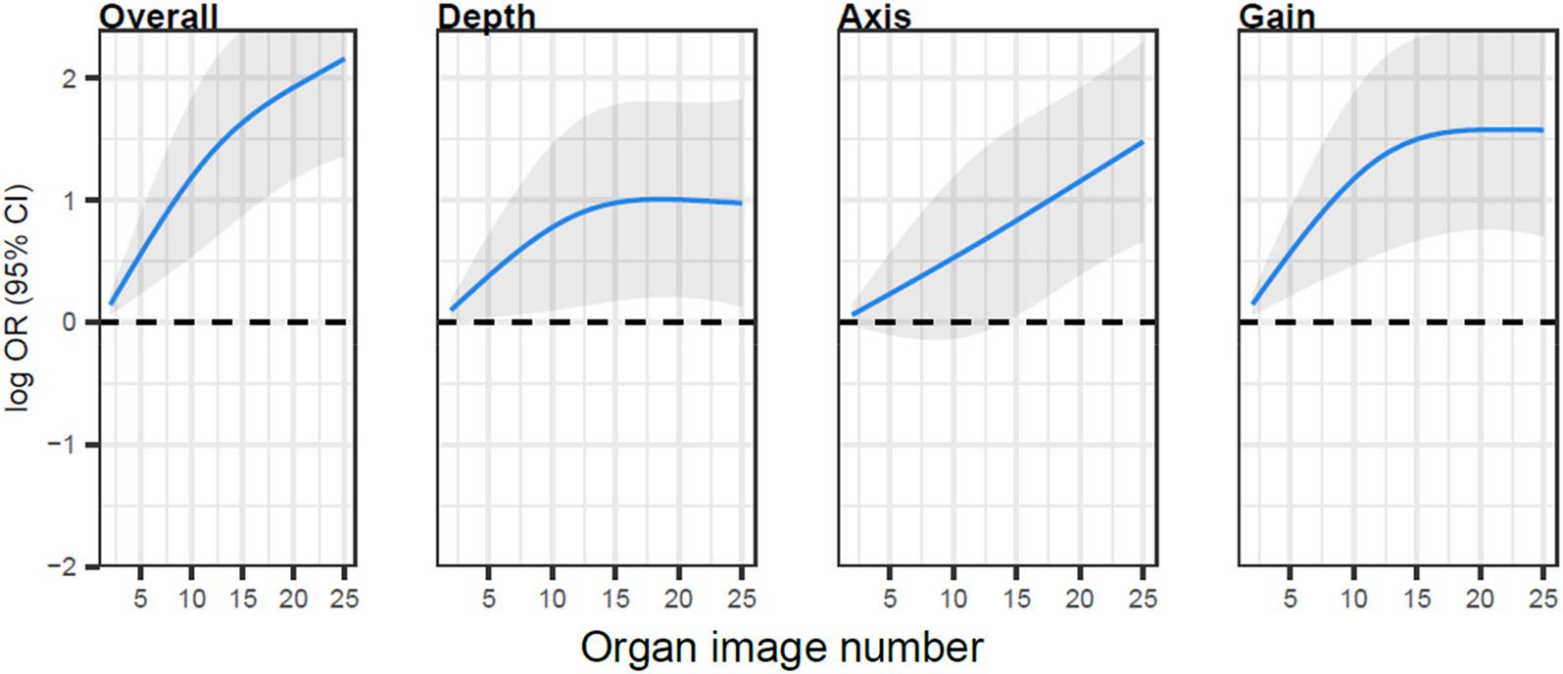
Learning curves for abdominal aorta point-of-care ultrasound examinations

For the abdominal aorta POCUS examination, nearly 80% of examinations received a 4 (28%) or 5 (51%) with less than 5% receiving a 1 or a 2 on the 5-point Likert scale for overall image quality (Table 1 and Fig. 1). Students’ scores generally improved over the first 15 to 20 exams, with a statistical plateau point after 19 examinations on aggregate analysis, with relatively less or no improvement following (Additional file 1: Fig. S1). Statistical plateau points were noted at14 for depth, and 15 for gain, however, persistent improvement without plateau was noted for axis (Additional file 1: Fig. S2).

**Fig. 2:**
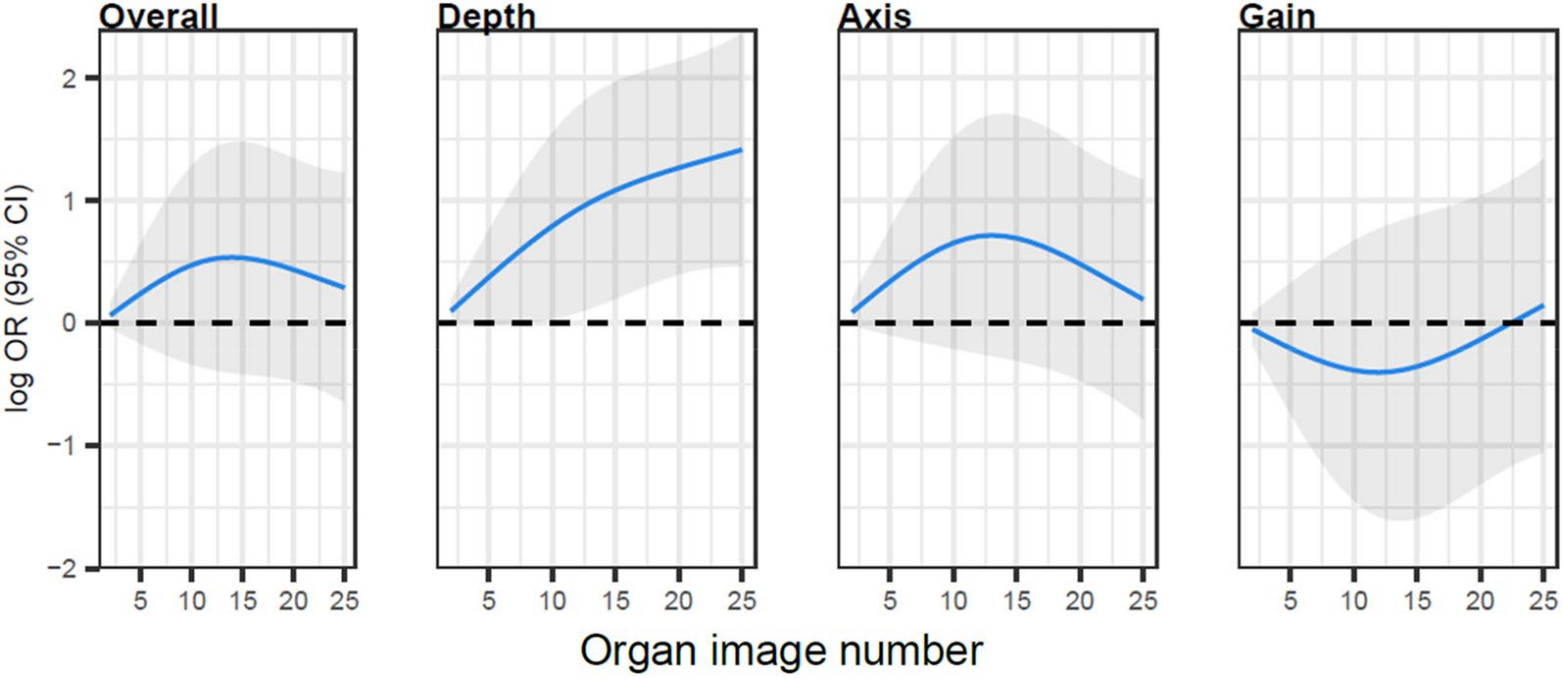
Learning curves for bladder point-of-care ultrasound examinations

For the bladder POCUS examination, cumulatively across the curriculum, 95% of examinations received a 4 (17%) or 5 (78%) on the 5-point Likert scale, with all other examinations being a 3 (Table 1 and Fig. 2). There was evidence of improvement over time in depth through the first 10 to 15 number of examinations, with a plateau point of 15 (Additional file 1: Fig. S3). Scores for overall image quality, axis, and gain tended to remain similar over time, without statistically significant plateau points (Additional file 1: Fig. S4).

**Fig. 3:**
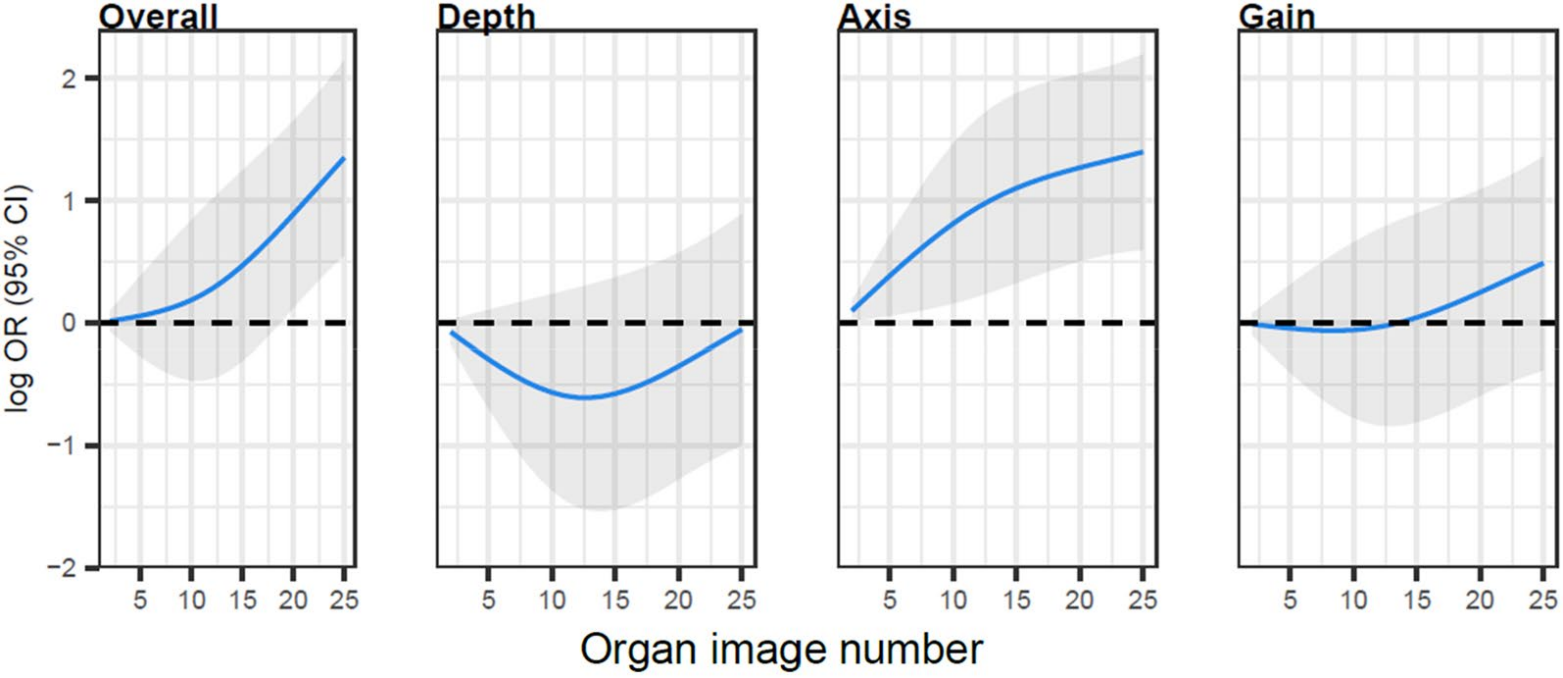
Learning curves for cardiac point-of-care ultrasound examinations

For the cardiac POCUS examination, cumulatively across the curriculum, 75% of examinations received a 4 (46%) or 5 (29%), with 4% receiving less than 3 on the 5-point Likert scale for overall image quality (Table 1 and Fig. 3). Students tended to improve for depth, gain, and overall image quality form the 10th to 25th examination, without statistically significant plateau points (Additional file 1: Fig. S5). Axis tended to improve through the first 15–20 examinations, with an aggregate plateau point of 17 (Additional file 1: Fig. S6).

**Fig. 4:**
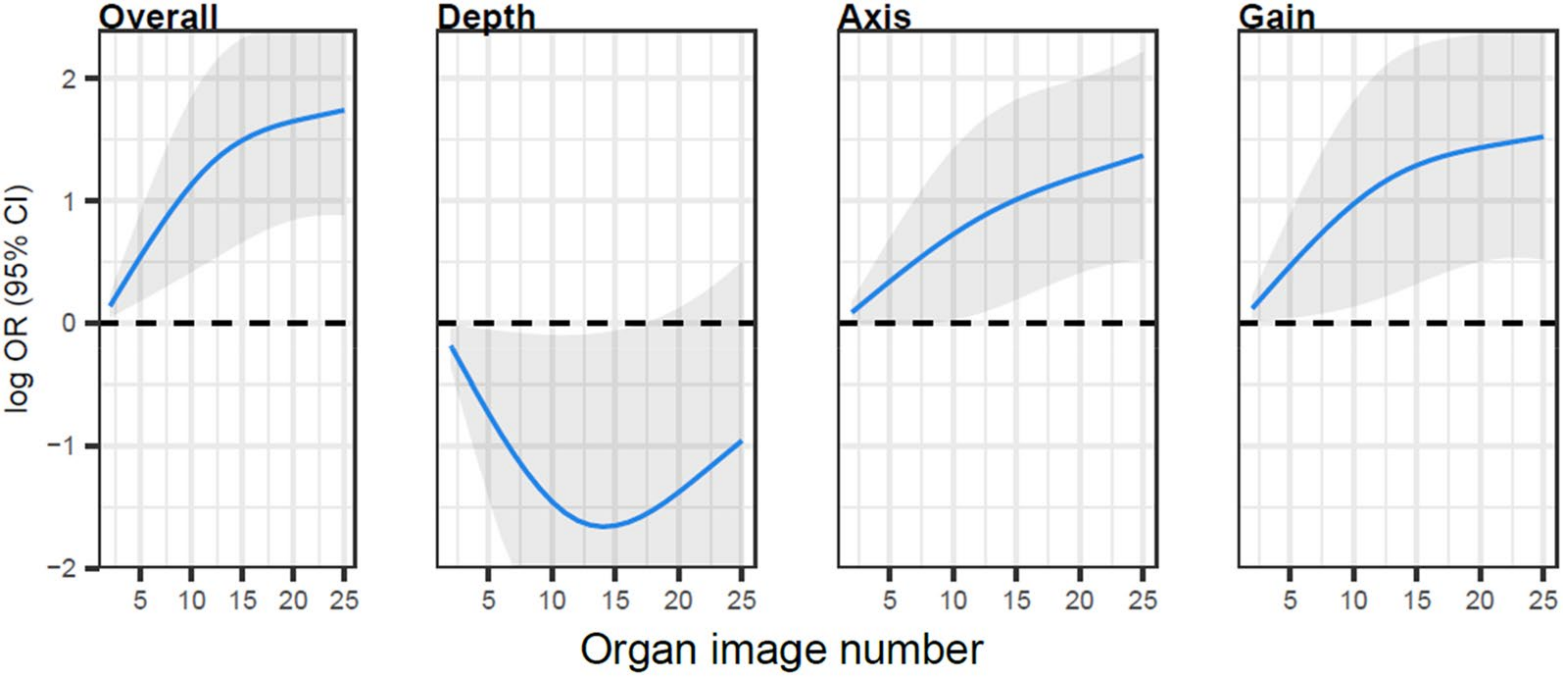
Learning curves for lung point-of-care ultrasound examinations

For the lung POCUS examination, cumulatively across the curriculum, 92% of examinations received a 4 (27%) or 5 (65%), with 8% receiving a 3 on the 5-point Likert scale for overall image quality (Table 1 and Fig. 4). Overall image quality, axis, and gain tended to improve over the first 10–15 exams, with aggregate plateau points of 16, 16, and 15, respectively (Additional file 1: Figs. S7 and S8). Scores for depth worsened over the first 10–12 exams with some evidence for improvement thereafter, with an aggregate plateau point of 20 (Additional file 1: Fig. S8).

**Fig. 5:**
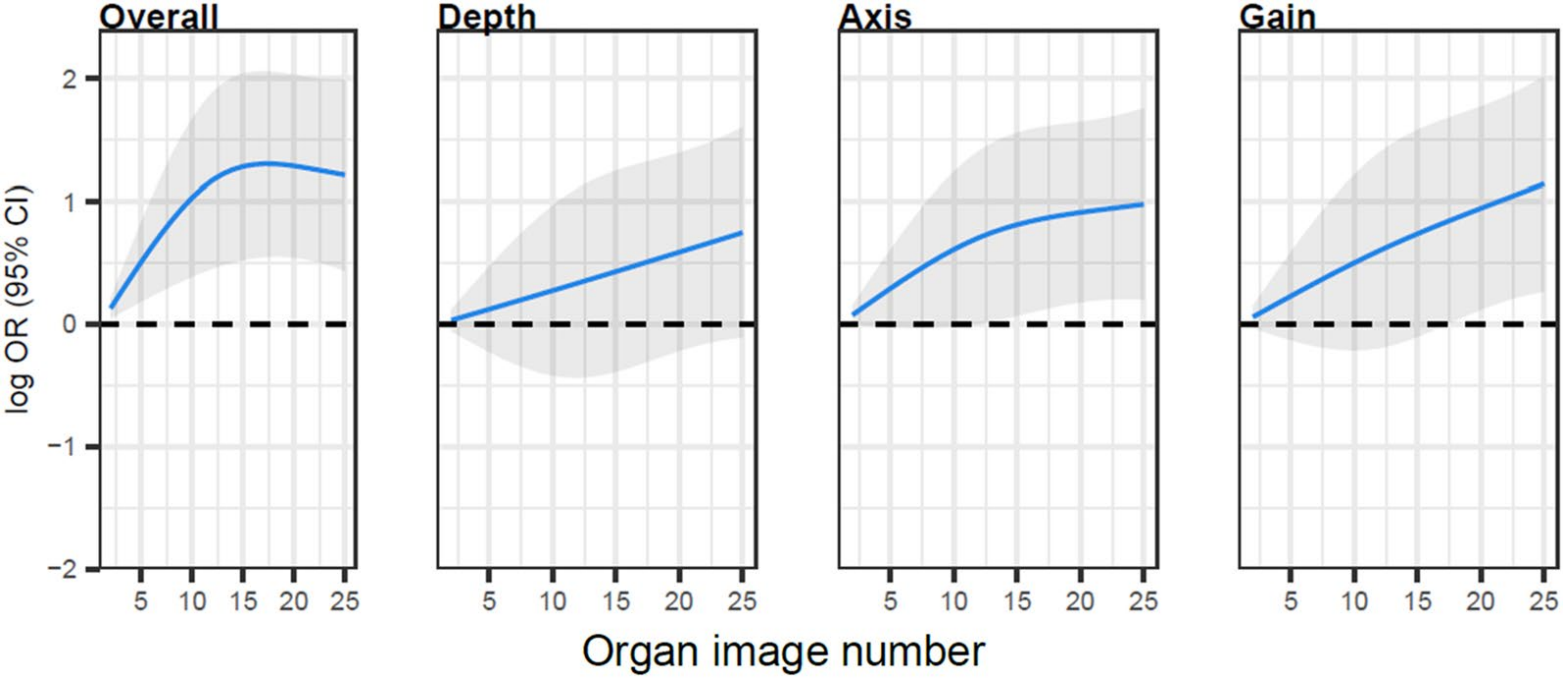
Learning curves for renal point-of-care ultrasound examinations

For the renal POCUS examination, cumulatively for the entirety of the curriculum, 83% of examinations received a 4 (36%) or 5 (47%), with 17% receiving a 3 on the 5-point Likert scale for overall image quality (Table 1 and Fig. 5). While overall image quality scores reached a plateau by the 15th image, scores for depth showed little improvement and score for axis and gain showed improvement around exams 8–15 (Additional file 1: Figs. S9 and S10).

## Discussion

Group-level learning curves average results across a cohort and provide useful information for educators to guide education interventions and assessments [1]. Plateaus on group-level learning curves represent where a cohort of learners, on average, are unlikely to improve further unless an educational intervention is applied. Despite their utility, group-level learning curves do not represent the learning of any particular individual, and individuals might benefit from further repetition beyond a group-level plateau [1]. As such, this paper avoids equating plateau points with competence. Rather, understanding typical learning curves and plateaus helps guide ideal timing for re-education for those needing to improve performance [1]. Additionally, the learning curves help guide the appropriate timing of holistic learner evaluations, such as an observed structured clinical encounter in education or workplace competency assessment in clinical practice, by organ system to determine which students have competency and which require further assistance [1]. Importantly, such an assessment would require assessment of both the psychomotor skills of image generation and the cognitive skills of image interpretation and clinical integration.

This data suggest that learning curves differ for specific POCUS applications, which is corroborated by prior literature [7–9]. Our data suggest that bladder POCUS has the quickest learning curve for novice learners. Abdominal aorta, lung, and renal exams have longer learning curves, that overall similar to each other (plateau points 19, 16, and 15, respectively). Cardiac POCUS had the longest learning curve, without a plateau point for overall image quality, suggesting ongoing improvement is noted beyond 25 examinations.

To our knowledge, learning curves for bladder ultrasound have not been previously defined. Bladder POCUS had the highest rate of exemplary image acquisition scores throughout the entire curriculum, with performance remaining stable over the course of the curriculum. This data suggest that bladder POCUS skill can be acquired rapidly for novice learners.

Data regarding learning curves for abdominal aorta POCUS demonstrate substantial heterogeneity. In assessment of military physicians, it was thought that 20 examinations were sufficient for a plateau of learning [12]. However, in a more robust analysis of emergency medicine physicians, Blehar demonstrated a plateau point of 84 examinations [7]. Importantly, in the study by Blehar et al. “learner image quality was assessed by comparing their image quality to the image quality of the expert reviewers’ independent imaging performed during the study”. Comparison to experts in the Blehar et al. likely prolonged the learning curves, where our data suggest that learning based on a standardized assessment plateaus much sooner. Similarly, data regarding lung POCUS have substantial variation. Blehar et al. found a plateau point of 39 lung examinations, but most other studies favor a steeper learning curve for lung ultrasound [7, 12, 14, 15]. Our study corroborates that psychomotor skill acquisition for the lung plateaus relatively quickly for novice learners.

Review of the literature shows that plateau points for cardiac POCUS occur after between 20 and 36 examinations [7–9, 21]. Importantly, Millington found a plateau point of 20 examinations in surgical residents with “low experience” with POCUS. However, residents were defined as being “low experience” despite completing up to 25 examinations prior to the study [9]. This likely represents an underestimation of true learning curves, given the learners’ prior experience. Blehar noticed a statistically defined plateau point after 27 cardiac examinations, however, on graphical representation improvement is continually noted after this point [7]. While assessing “entrustability” of POCUS skills, Clunie found that predefined entrustability was met after an average of 36 exams [8]. Our study would suggest that cardiac POCUS is the most difficult application of those studied within this cohort to learn, and that learning might continue beyond 25 examinations. Educational and evaluation measures to ensure adequate learning should be designed with this knowledge in mind.

Depth, tomographic axis, and gain are important components of image quality [4]. Analysis of rates of scores (1–5 on the Likert score) and learning curves for depth, tomographic axis, and gain demonstrates that tomographic axis has the longest learner curve of the three components of image quality for this cohort of learners. Visually, the components of depth and gain were noted to have higher rates of exemplary scores earlier on in learning than when compared to axis (supplemental digital content 3–7). This is corroborated by learning curves, within specific organs. For example, statistical plateau points for abdominal aorta were noted at 19 for overall image quality, 14 for depth, and 15 for gain; however, ongoing improvement without plateau was noted for tomographic axis. Regarding cardiac imaging, the combination of higher rates of exemplary scores on depth and gain, combined with a lack of statistical improvement over time suggest students were able to rapidly acquire these skills. However, data for cardiac axis demonstrate worse performance overall, and a plateau point at 17 examinations. This would suggest that tomographic axis was the hardest to master. Overall, this would suggest that tomographic axis was the hardest concept of the three for this cohort of learners to grasp. One potential conclusion is that the concepts of depth and gain are more generalizable between organ systems, while an individual organ’s tomographic axis is unique. This difference between components of image quality has not been reported in the literature previously. While teaching all principles of image quality is important, this data suggest that curricular changes paying particular attention to optimizing the unique tomographic axis for each organ system might benefit our program. Further research is required to see if this is finding can be generalized to other learners.

Limitations to this study include it being from a single cohort of students within a specific educational program. While students were encouraged to save all examinations within their Butterfly Network archive, it is likely that some studies over the course of the curriculum were not saved by students, and thus not reviewed. This potentially impacts learning curves and plateau points, however, given the requirements of the students to complete a portfolio of images it is unlikely that this would represent a significant number of missed examinations. While all students completed the required minimum number of exams, incomplete capture of data is noted during the image review and data exportation phases of the research project, as some students had fewer than 25 examinations per organ system in the extracted data. We believe this represents a small subset of students, with minimal impact on overall data analysis. Lastly, while practically there is benefit of learning the psychomotor skill component on healthy individuals, this study does not address learning curves on, or transfer of skill to actual patients in the clinical environment.

Further research incorporating multiple sites and educational programs is needed to identify more generalizable data. It stands to reason that the quality and amount of education on a specific topic will affect learning curves. However, studies evaluating the impact of instructional design on learning curve do not exist currently. Studies assessing the effect on learning curves of the amount of education, the type of evaluation, and the frequency of feedback should be completed. Additionally, research to assess transfer of skills from practicing on healthy individuals to actual patients is required to determine optimal amounts of practice in preclinical education.

## Conclusion

This research shows that novice learners acquire psychomotor skills for POCUS rapidly, however, the rate of skill acquisition is organ dependent. Tomographic axis is the hardest component of image quality to learn, potentially because it is less generalizable. These insights into the variable rates of skill acquisition between organs, and between components of image quality provide valuable insights for educators designing and implementing a curriculum.

## Supplementary Information

**Additional file 1: Figure S1.** Stacked bar charts of proportion of scores on a 5-Point Likert scale based on number of abdominal aorta examinations. **Figure S2.** Plateau Points for Abdominal Aorta Point-of-Care Ultrasound examinations. **Figure S3.** Plateau Points for Bladder Point-of-Care Ultrasound examinations. **Figure S4.** Stacked bar charts of proportion of scores on a 5-Point Likert scale based on number of bladder examinations. **Figure S5. **Stacked bar charts of proportion of scores on a 5-Point Likert scale based on number of cardiac examinations. **Figure S6.** Plateau Points for Cardiac Point-of-Care Ultrasound examinations. **Figure S7.** Stacked bar charts of proportion of scores on a 5-Point Likert scale based on number of lung examinations. **Figure S8.** Plateau Points for Lung Point-of-Care Ultrasound examinations. **Figure S9.** Stacked bar charts of proportion of scores on a 5-Point Likert scale based on number of renal examinations. **Figure S10.** Plateau Points for Renal Point-of-Care Ultrasound examinations.

## Data Availability

Unable to be shared publicly.
