# Supplementary material for: Learning curves for point-of-care ultrasound image acquisition for novice learners in a longitudinal curriculum
Source: Ultrasound J. 2023 Jul 5;15:31. doi: 10.1186/s13089-023-00329-2 (PMC10319692; doi:10.1186/s13089-023-00329-2)

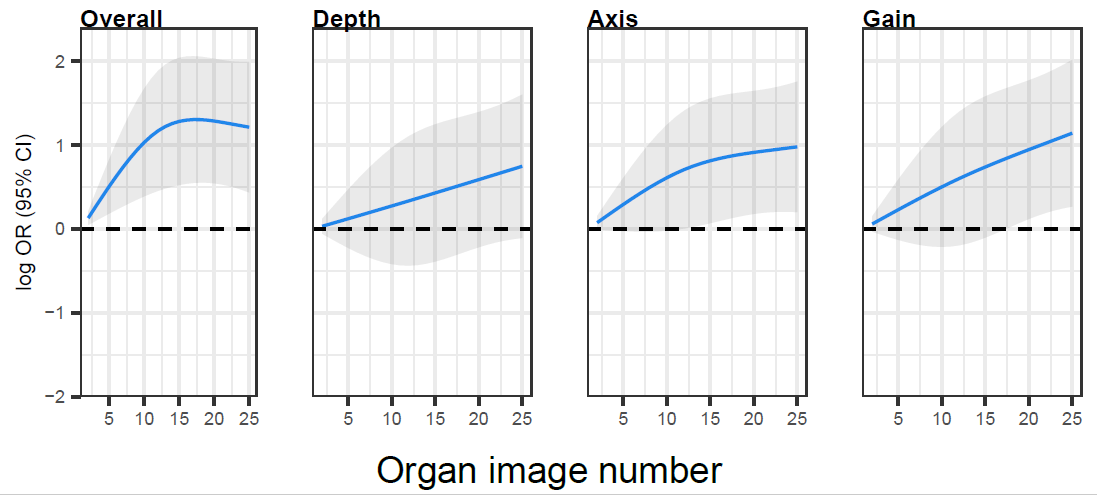


**Additional file 1: Figure S1.** Stacked bar charts of proportion of scores on a 5-Point Likert scale based on number of abdominal aorta examinations


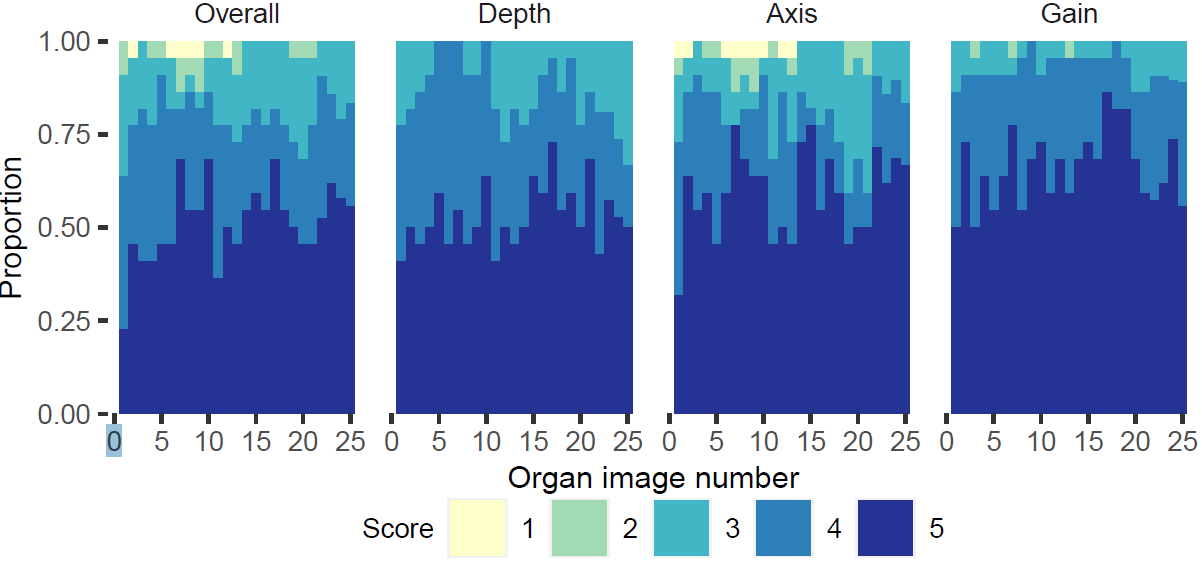


**Additional file 1: Figure S2.** Plateau Points for Abdominal Aorta Point-of-Care Ultrasound examinations


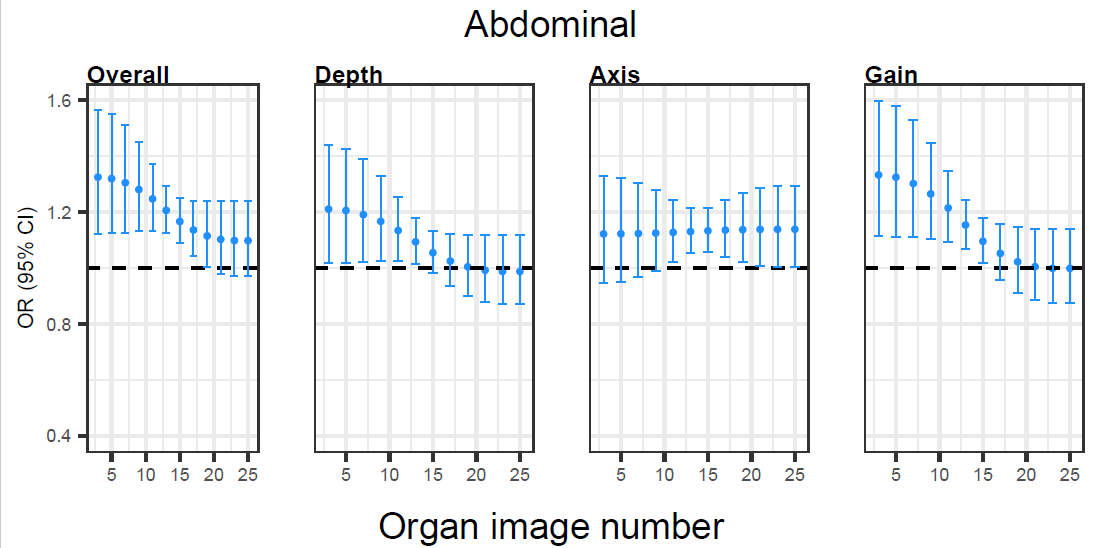


**Additional file 1: Figure S3.** Plateau Points for Bladder Point-of-Care Ultrasound examinations


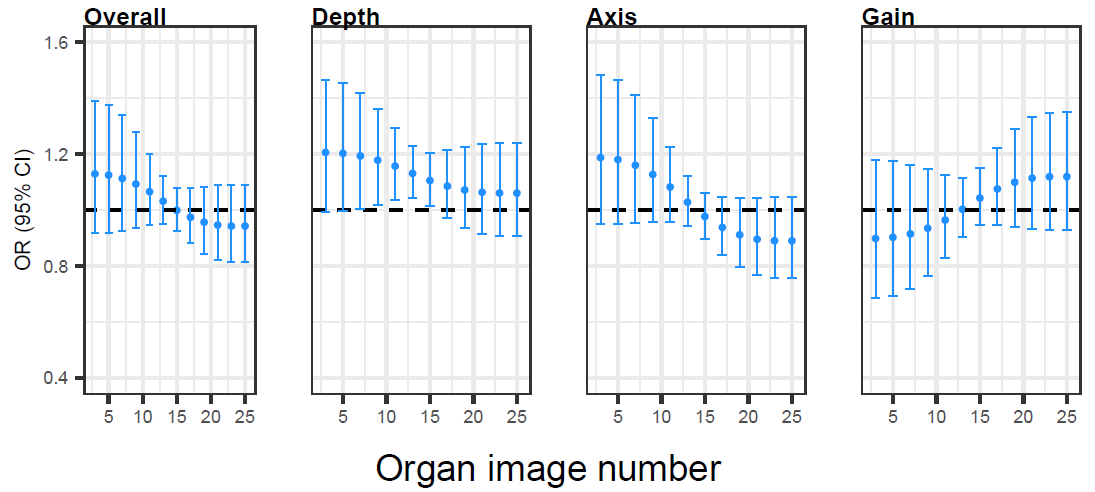


**Additional file 1: Figure S4.** Stacked bar charts of proportion of scores on a 5-Point Likert scale based on number of bladder examinations


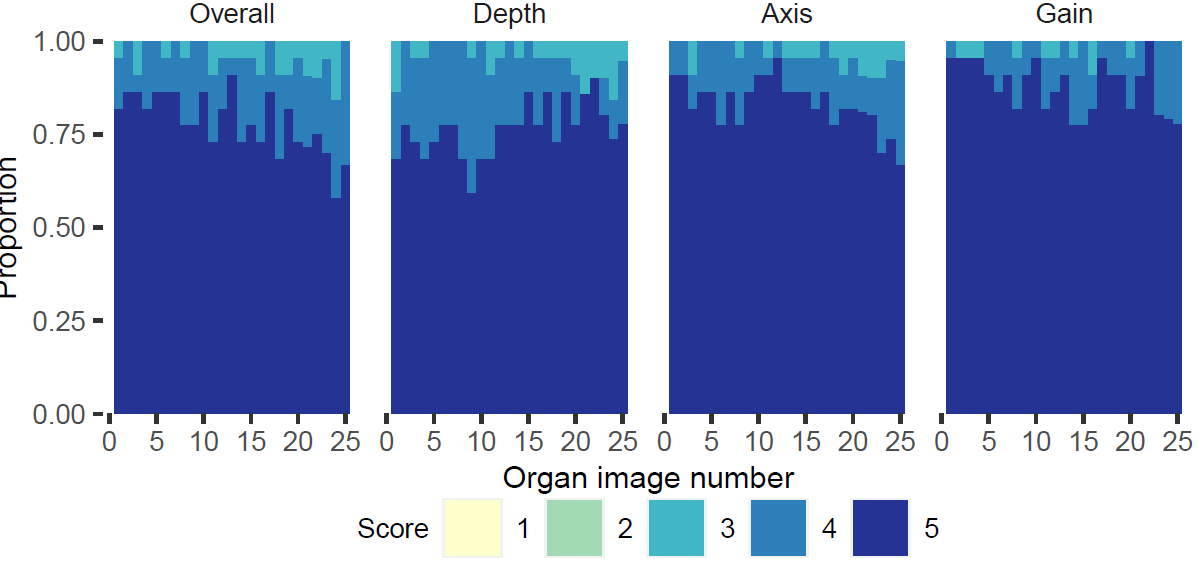


**Additional file 1: Figure S5.** Stacked bar charts of proportion of scores on a 5-Point Likert scale based on number of cardiac examinations.


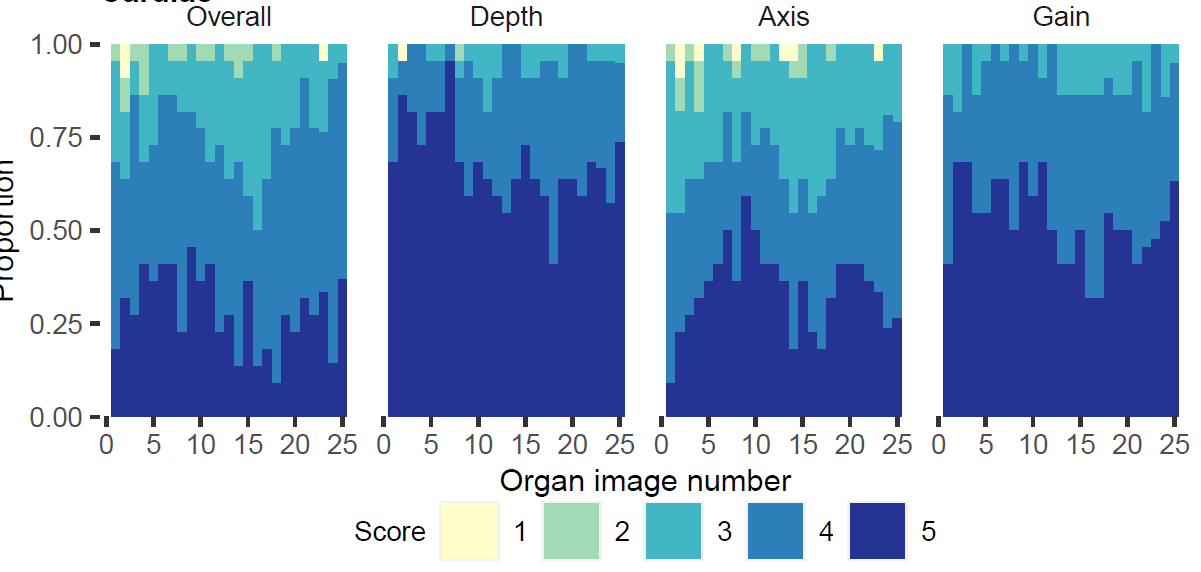


**Additional file 1: Figure S6.** Plateau Points for Cardiac Point-of-Care Ultrasound examinations


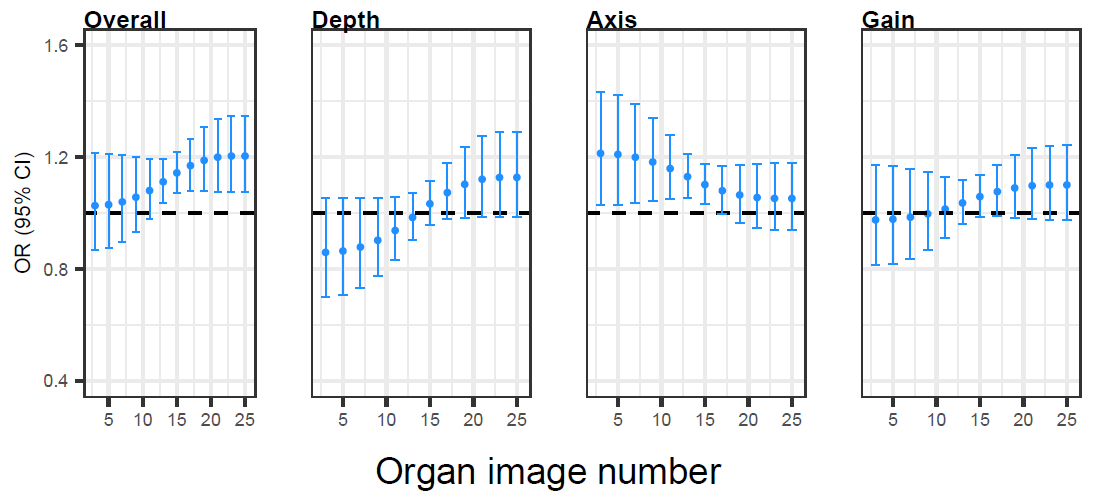


**Additional file 1: Figure S7.** Stacked bar charts of proportion of scores on a 5-Point Likert scale based on number of lung examinations


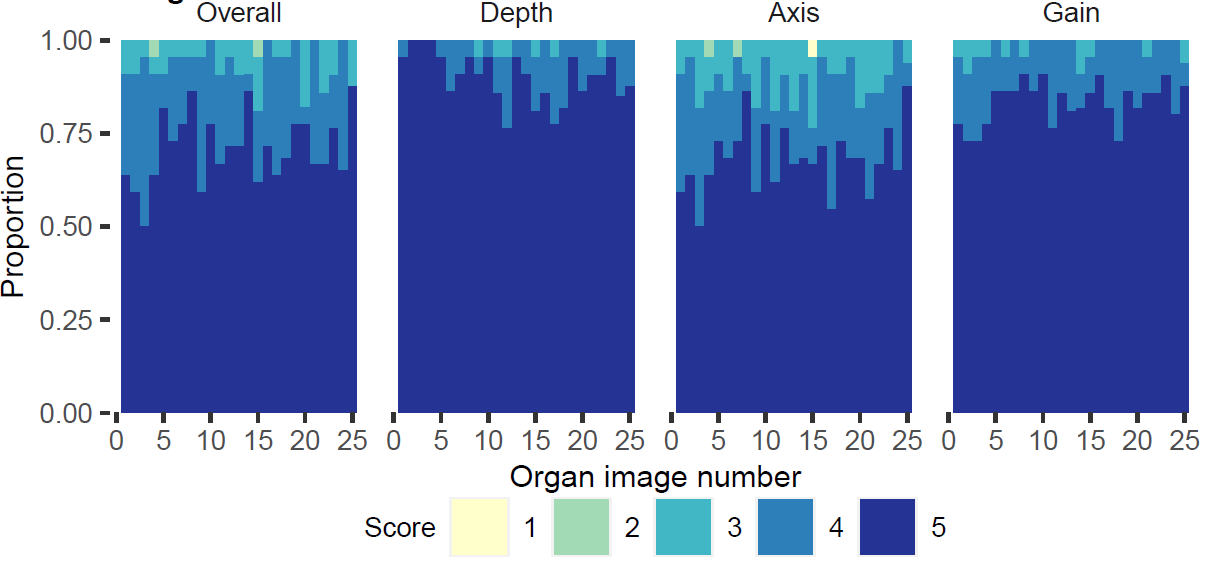


**Additional file 1: Figure S8.** Plateau Points for Lung Point-of-Care Ultrasound examinations


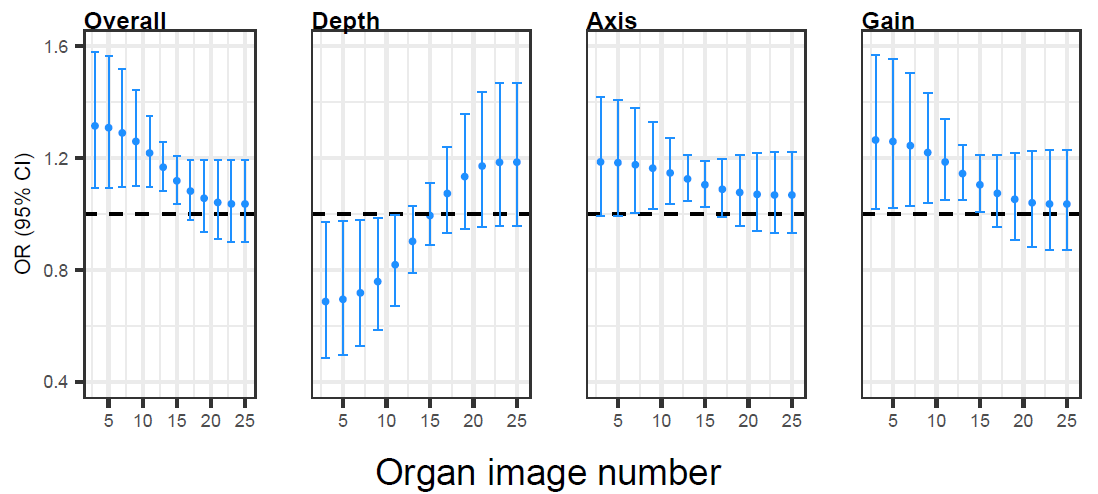


**Additional file 1: Figure S9.** Stacked bar charts of proportion of scores on a 5-Point Likert scale based on number of renal examinations.


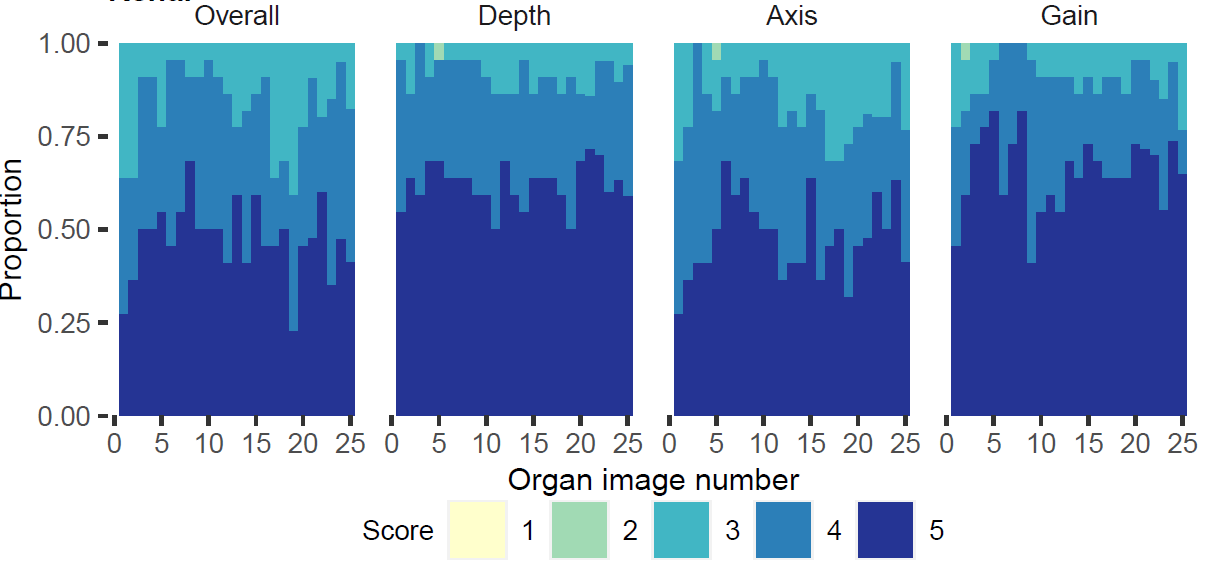


**Additional file 1: Figure S10.** Plateau Points for Renal Point-of-Care Ultrasound examinations


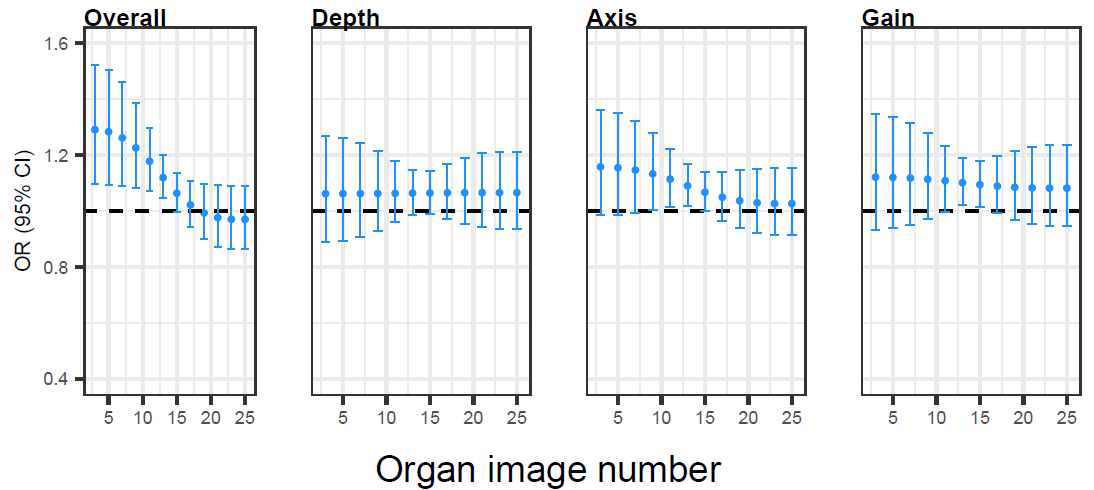

Supplement: Supplementary file 1 — Additional file 1: Figure S1. Stacked bar charts of proportion of scores on a 5-Point Likert scale based on number of abdominal aorta examinations. Figure S2. Plateau Points for Abdominal Aorta Point-of-Care Ultrasound examinations. Figure S3. Plateau Points for Bladder Point-of-Care Ultrasound examinations. Figure S4. Stacked bar charts of proportion of scores on a 5-Point Likert scale based on number of bladder examinations. Figure S5. Stacked bar charts of proportion of scores on a 5-Point Likert scale based on number of cardiac examinations. Figure S6. Plateau Points for Cardiac Point-of-Care Ultrasound examinations. Figure S7. Stacked bar charts of proportion of scores on a 5-Point Likert scale based on number of lung examinations. Figure S8. Plateau Points for Lung Point-of-Care Ultrasound examinations. Figure S9. Stacked bar charts of proportion of scores on a 5-Point Likert scale based on number of renal examinations. Figure S10. Plateau Points for Renal Point-of-Care Ultrasound examinations. [file 13089_2023_329_MOESM1_ESM.docx]
